# Insulin Glargine and Cancer Risk in Patients with Diabetes: A Meta-Analysis

**DOI:** 10.1371/journal.pone.0051814

**Published:** 2012-12-19

**Authors:** Xulei Tang, Lin Yang, Zhiyu He, Jingfang Liu

**Affiliations:** 1 Department of Endocrinology, the First Hospital of Lanzhou University, Lanzhou, Gansu, China; 2 Department of Internal Medicine, the First Clinical Medical School, Lanzhou University, Lanzhou, Gansu, China; Tehran University of Medical Sciences, Iran (Islamic Republic of)

## Abstract

**Aim:**

The role of insulin glargine as a risk factor for cancer is controversial in human studies. The aim of this meta-analysis was to evaluate the relationship between insulin glargine and cancer incidence.

**Methods:**

All observational studies and randomized controlled trials evaluating the relationship of insulin glargine and cancer risk were identified in PubMed, Embase, Web of Science, Cochrane Library and the Chinese Biomedical Medical Literature Database, through March 2012. **Odds ratios (ORs)** with corresponding 95% confidence interval (CI) were calculated with a random-effects model. Confidence in the estimates of the obtained effects (quality of evidence) was assessed by using the Grading of Recommendations Assessment, Development, and Evaluation approach.

**Results:**

A total of 11 studies including 448,928 study subjects and 19,128 cancer patients were finally identified for the meta-analysis. Insulin glargine use was associated with a lower **odds** of cancer compared with non-glargine insulin use (**OR 0.81, 95% CI 0.68 to 0.98, P = 0.03;** very low-quality evidence). Glargine did not increase the odds of breast cancer (**OR 0.99, 95% CI 0.68 to 1.46, P = 0.966**; very low-quality evidence). Compared with non-glargine insulin, no significant association was found between insulin glargine and **prostate cancer, pancreatic cancer and respiratory tract cancer.** Insulin glargine use was associated with lower **odds** of **other site-specific cancer.**

**Conclusions:**

Results from the meta-analysis don't support the link between insulin glargine and an increased risk of cancer and the confidence in the estimates of the effects is very low. Further studies are needed to examine the relation between insulin glargine and cancer risk, especially breast cancer.

## Introduction

Diabetes mellitus has become a significant health care problem throughout the world. From a survey of the International Diabetes Federation, there are 366 million people with diabetes in 2011, and the total number is expected to rise to 552 million by 2030 [1]. **Type 1 diabetes accounts for 5%–10% of the total cases of diabetes and type 2 diabetes accounts for 90%–95%** [**2**] **.** Diabetes is a progressive disorder and associated with serious complications and increased mortality. The most important goal in the treatment of patients with diabetes is to lower the risk of diabetic complications. Glucose-lowering therapy is the first step to prevent diabetic complications and reduce mortality. **Type 1 diabetes requires insulin therapy in the beginning.** For patients with type 2 diabetes, most patients are initially treated with oral hypoglyceimic agents, but every available oral hypoglycaemic agent has limited glucose-lowering efficacy because of the progressive loss of pancreatic beta-cell function and decreased insulin sensitivity. Therefore, **half of** patients eventually require insulin therapy to achieve the ideal glycemic control targets.

Insulin glargine, a long-acting recombinant human insulin analog with only injected once-daily, induces a smooth metabolic effect that lasts for at least 24 hours with no pronounced peak [3]. **It differs from human insulin by replacing asparagine with glycine in position 21 of the A-chain and by carboxy-terminal extension of B-chain by 2 arginine residues. Insulin glargine is recommended to patients with diabetes who attempt to improve glycemic control while avoiding severe and nocturnal hypoglycemia and it provides a safer basal insulin supply than neutral protamine hagedorn insulin because of the smooth metabolic effect that lasts for at least 24 hours with no pronounced peak** [4]. However, in 2009, four remarkable papers [5]–[8] that linked insulin glargine with a putative increased risk of cancer incidence were simultaneously published in Diabetologia, which aroused an unprecedented controversy about cancer risk profile of insulin glargine [9]. These four observational studies also have generated contrasting results and led to considerable insecurity of patients treated with insulin glargine. Then later, not unexpectedly, many researchers began to explore their databases to seek evidence for the potential relationship of insulin glargine and an increased incidence of cancer. However, these studies served only to perpetuate the inconclusiveness [10].

**Some in vitro data showed that the mitogenic potency of insulin glargine was higher compared with human insulin, regular insulin and other insulin analogue in vitro** **[11]**, **[12]** **. This may represents one potential mechanism contributing to progression of cancer. Others showed that the mitogenic potency of insulin glargine was similar to human insulin** [13]–[15].

Therefore, we performed a meta-analysis to evaluate if the use of insulin glargine increases risk of cancer incidence.

## Materials and Methods

To avoid bias the methods for post hoc analysis and inclusion criteria were specified in advance and protocol-defined. **The study was performed in accordance with the Quality of Reporting of Meta-analysis (PRISMA, MOOSE) guidelines** [**16]**, **[17]** **.**

### Search strategy and study selection

All studies (from the beginning of indexing for each database to March 12, 2012) evaluating the relationship between insulin glargine and cancer risk were initially searched using the **“insulin glargine”, “lantus”, “tumor”, “tumors”, “cancer”, “cancers” “neoplasm”, “neoplasms”and “malignancy” (Supplementary Data S1)** from five electronic search engines: PubMed, Embase, Web of Science, Cochrane Library and the Chinese Biomedical Medical Literature Database by two independent investigators (L. Y. and Z. H.). **In addition, manual search of other resources (including references from selected studies) and the search on Google Scholar were also carried out to identify more related articles.** No language restriction was imposed.

A study was included in the meta-analysis if it satisfied the following inclusion criteria: 1) all observational studies and randomized controlled trials (RCTs) evaluating the relationship of insulin glargine and cancer risk in patients with diabetes mellitus; 2) observational studies with insulin glargine and non-glargine insulin as exposure, and for RCTs insulin glargine was the treatment arm and non-glargine insulin was the comparator; **3) published in peer-reviewed journals in full-text form. 4) providing any of the following outcomes: overall cancer incidence and/or site-specific cancers incidence including breast cancer, prostate cancer, pancreatic cancer, gastrointestinal cancer, colorectal cancer, bladder cancer, respiratory tract cancer and hepatobiliary cancer.**

The investigators independently determined every eligible article for inclusion in the meta-analysis and resolved disagreements by discussion or consensus of a third reviewer (X. T.). If the same result was published in multiple reports, only the latest study was included in the meta-analysis.

### Data extraction and quality assessment

The two investigators independently extracted data from each included article. Discrepancies were resolved by discussion or involving the third investigator. The following information was abstracted on first author's surname, publication year, country where the data was obtained, study design, gender, the age of participant at studied insulin initiation, study population, the type of comparator, duration of follow-up, diagnostic method of cancer and outcomes.

The two investigators assessed the confidence in the estimates of effect of the body of evidence (quality of evidence) by outcome and produced the draft evidence profiles according to GRADE (Grading of Recommendations Assessment, Development, and Evaluation) system (http://www.gradeworkinggroup.org; last accessed March 29, 2012) [18], [19]. The completed evidence summaries and GRADE assessments were discussed by all of investigators. The confidence in the estimate of effect is categorized into 4 levels: high, moderate, low, and very low [20]. **RCTs rate the highest on the GRADE system and observational studies rate low. Five reasons that rate down the confidence in the estimate of effect include risk of bias, imprecision, indirectness, inconsistency, publication bias. Three reasons that rate up the quality of evidence include dose-response gradient, magnitude of effect, and issues of residual plausible confounding**. Evidence summaries were prepared for each outcome by using the GRADEpro 3.6 (McMaster University, Hamilton, Ontario, Canada).

### Statistical analysis

We performed quantitative analysis of individual study data using standard statistical procedures provided in STATA 12.0 (**stata**, College Station, TX, USA). The **odds ratios (ORs)** and corresponding 95% confidence intervals (CI) for each outcome were calculated using random-effects models. Statistical heterogeneity among studies was assessed using the chi-square test (results were defined as heterogeneous for a P value<0.10) [21], and was quantified through the I^2^ statistic [22]. Value of the I^2^ statistic equal to 0% indicates no observed heterogeneity and that >50% indicates substantial heterogeneity. **Potential publication bias was examined by Begg's test and Egger's test analysis. Two-sided tests were used with** P value<0.05 considered to be statistically significant except where otherwise specified.

As a primary analysis, the summary OR with the corresponding 95% CI of overall cancer for insulin glargine users versus non-glargine insulin users was estimated. Then subgroup analysis was performed according to comparators (including human insulin users, other insulin analogues users and insulin isophane users). We performed sensitivity analysis by limiting to observational studies and limiting to studies that excluded the patients with a history of any cancer before cohort entry. **We also conducted sensitivity analysis according to the type of observational studies and the different source of data on insulin glargine therapy use.** In secondary analyses, the estimates of site-specific cancers, including breast cancer, colorectal cancer, prostate cancer, pancreatic cancer, gastrointestinal cancer, bladder cancer, respiratory tract cancer and hepatobiliary cancer, were calculated for insulin glargine users versus non-insulin glargine users. Sensitivity analyses were performed in breast cancer by limiting to observational studies and limiting to studies that excluded the patients with cancer history before cohort entry.

## Results

### Identified studies

**Figure 1:**
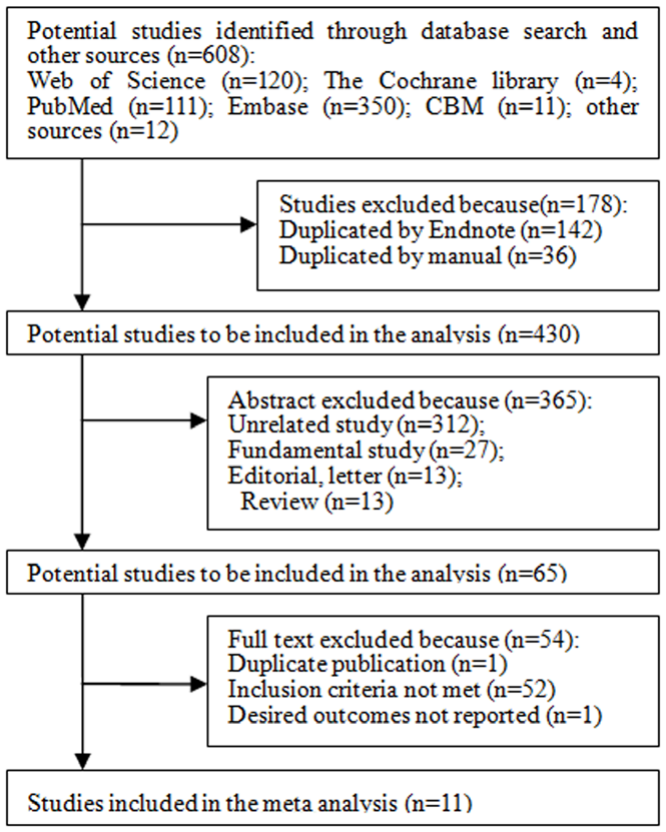
Flow diagram of study selection process. CBM, the Chinese Biomedical Medical Literature Database.

A detailed flow diagram of the study selection for the meta-analysis is presented in Figure 1. A total of 608 potentially related studies were identified via the search strategy listed in previous section. After finding duplicates and reviewing the titles, abstracts and full texts, 11 studies including 448, 928 study subjects and 19, 128 cancer patients were finally identified for the meta-analysis [7], [8], [23]–[31]. The study design consisted of **1 RCT** [**31**] **and 10 observational studies (1 case-control study** [**30**] **and 9 cohort studies** [**7]**, [8], [23]–**[29]** **)**. The data were obtained from ten countries: Netherland, France, UK, USA, Sweden, China, Italy, Canada, Germany and Scotland. **The study by Mannucci et al** [**30**] **used self-reported and prescription record data on insulin glargine therapy use, others used prescription record data** [**7]**, [8], [23]–[29], **[31]** **.** Only 8 studies [7], [8], [23], [24], [26]–[29] adjusted for confounders, such as age, sex, type of diabetes, comorbidities and concomitant drug (Table 1). **A study** [**26**] **that excluded patients with a history of breast cancer only reported the association between insulin glargine and the risk of breast cancer, but not report relative risk of insulin glargine and overall cancer.** Two studies [7], [27] included some patients with a history of cancer before cohort entry. The main baseline characteristics of the included studies are reported in Table 1. Table 2 summarizes the findings and the quality of the evidence for insulin glargine compared with non-glargine insulin therapy.

**Table 1:** The main baseline characteristics of included studies.

Study	Country	Study design	The method of obtaining drug exposure	Subject (percent of male)	Comparator	Mean Age (years)		Duration of follow-up (years)		Covariates
						IG	NGI	IG	NGI	
Ruiter 2012 [23]	Netherland	Prospective cohort	Prescription record	19337 (47.86%)	Human insulin, other insulin analogues	63.1	63.8	2.26	3.67	Age, sex, calendar time, hospitalization, unique drugs, other insulin use
Blin 2012 [24]	France	Prospective cohort	prescription record	1843 (46.99%)	Human insulin	67.8	69.9	NR	NR	Sex, type of diabetes, age, comorbidities, concomitant drug
van Staa 2011 [25]	UK	Prospective cohort	prescription record	36738(55.80%)	Other insulin analogues, insulin isophane	NR	NR	3.00	3.80	None
Suissa 2011 [26]	UK	Prospective cohort	prescription record	15227 (0%)	Non-glargine insulin	62.9	62.2	NR	NR	Age, excessive alcohol use, smoking status, obesity, HbA1c, diabetes and insulin use duration, cancer history, oophorectomy, HRT use, sulfonylureas thiazolidinediones, metformin and statins
Morden 2011 [27]	USA	Retrospective cohort	prescription record	66400 (31.26%)	Non-glargine insulin	76.9	77.6	1.98	1.93	Age, race, diabetes complications, obesity, estrogen use, tobacco, income, comorbidities and insulin dose
Ljung 2011 [28]	Sweden	Prospective cohort	prescription record	94523 (56.79%)	Non-glargine insulin	NR	NR	3.00	3.00	Age, sex
Chang 2011 [29]	Taiwan	Retrospective cohort	prescription record	53315 (48.01%)	Human insulin	60.65	62.07	1.48	2.10	Age, initiation year, sex, complication, concomitant drug, timing-varying medication use, dosage of insulin
Mannucci 2010 [30]	Italy	case-control	self-report	482 (51.66%)	Human insulin, other insulin analogues	68.9	68.0	6.33	6.33	None
Rosenstock 2009 [31]	USA,									
Canada	RCT	prescription record	1017 (53.88%)	Insulin isophane	54.9	55.3	4.29	4.28	None	
Hemkens 2009 [8]	Germany	Prospective cohort	prescription record	127031 (42.13%)	Human insulin, other insulin analogues	69.5	69.4	1.31	1.70	Age, sex, dose, oral glucose-lowering agents, concomitant medication, federal state, year, hospitalization
Colhoun 2009 [7]	Scotland	Prospective cohort	prescription record	32742 (53.01%)	Non-glargine insulin	68*	55*	NR	NR	prior cancer, type of diabetes, calendar year, sex,age, oral hypoglycaemic drugs, diabetes duration, HbA1c, diastolic BP, systolic BP and deprivation quintile, smoking, BMI

BP = blood pressure; HRT = Hormone replacement therapy; IG = insulin glargine; NGI = non-glargine insulin; NR = not reported; RCT = randomized controlled trial.

* median.

**Table 2:** GRADE Evidence Profile for insulin glargine versus non-glargine insulin.

Outcome	Participants (studies)	Overall quality of evidence	Study event rates, n/N (%)		Relative effect (95% CI)	Anticipated absolute effects	
			With Non-glargine insulin	With Insulin glargine		Risk with Non-glargine insulin	Risk difference with Insulin glargine (95% CI)
Overall cancer	433701 (10 studies) [7], [8], [23]–[25], [27]–[31]	⌖⊝⊝⊝VERY LOW*,†due to risk of bias, inconsistency	15979/363228 (4.4%)	2903/70473 (4.1%)	OR 0.81 (0.68 to 0.98)	44 per 1000	8 fewer per 1000 (from 1 fewer to 14 fewer)
Breast cancer	284402 (8 studies) [7], [23], [24], [26]–[29], [31]	⌖⊝⊝⊝VERY LOW*,†,‡due to risk of bias, inconsistency, imprecision	1104/241976 (0.46%)	260/42426 (0.61%)	OR 0.99 (0.68 to 1.46)	5 per 1000	0 fewer per 1000 (from 1 fewer to 2 more)
Colorectal cancer	268160 (6 studies) [7], [23], [24], [27]–[29]	⌖⊝⊝⊝VERY LOW*,§due to risk of bias	1268/230827 (0.55%)	148/37333 (0.4%)	OR 0.69 (0.56 to 0.85)	5 per 1000	1 fewer per 1000 (from 1 fewer to 2 fewer)
Prostate cancer	268160 (6 studies) [7], [23], [24], [27]–[29]	⌖⊝⊝⊝VERY LOW*,‡,§due to risk of bias, imprecision	1025/230827 (0.44%)	184/37333 (0.49%)	OR 0.94 (0.63 to 1.42)	4 per 1000	0 fewer per 1000 (from 2 fewer to 2 more)
Pancreatic cancer	285561 (6 studies) [7], [24], [25], [27]–[29]	⌖⊝⊝⊝VERY LOW*,§,∥due to risk of bias, imprecision	483/243380 (0.2%)	115/42181 (0.27%)	OR 1.08 (0.8 to 1.44)	2 per 1000	0 more per 1000 (from 0 fewer to 1 more)
Gastrointestinal cancer	148855 (3 studies) [28], [29], [31]	⌖⊝⊝⊝VERY LOW*,§,¶due to risk of bias, imprecision	699/133329 (0.52%)	45/15526 (0.29%)	OR 0.7 (0.51 to 0.95)	5 per 1000	2 fewer per 1000 (from 0 fewer to 3 fewer)
Bladder cancer	75512 (4 studies) [23], [24], [29], [31]	⌖⊝⊝⊝VERY LOW*,§,¶due to risk of bias, imprecision	166/61028 (0.27%)	24/14484 (0.17%)	OR 0.6 (0.37 to 0.99)	3 per 1000	1 fewer per 1000 (from 0 fewer to 2 fewer)
Respiratory tract cancer	108254 (5 studies) [7], [23], [24], [29], [31]	⌖⊝⊝⊝VERY LOW*,§,¶due to risk of bias, imprecision	410/93323 (0.44%)	58/14931 (0.39%)	OR 0.91 (0.59 to 1.41)	4 per 1000	0 fewer per 1000 (from 2 fewer to 2 more)
Hepatobiliary cancer	56175 (3 studies) [24], [29], [31]	⌖⊝⊝⊝VERY LOW*,§,¶due to risk of bias, imprecision	408/45480 (0.9%)	44/10695 (0.41%)	OR 0.51 (0.37 to 0.70)	9 per 1000	4 fewer per 1000 (from 3 fewer to 6 fewer)

* Only some studies presenting effect estimates adjusted for known confounders.

† High heterogeneity among studies.

‡ 95% confidence interval around the pooled includes both 1) no effect and 2) appreciable benefit and appreciable harm.

§ Although we did not downgrade, publication bias cannot be excluded.

∥ 95% confidence interval around the pooled includes both 1) no effect and 2) appreciable harm.

¶ 95% confidence interval around the pooled includes both 1) no effect and 2) appreciable benefit.

### Quantitative findings

#### Insulin glargine and overall cancer incidence

**Figure 2:**
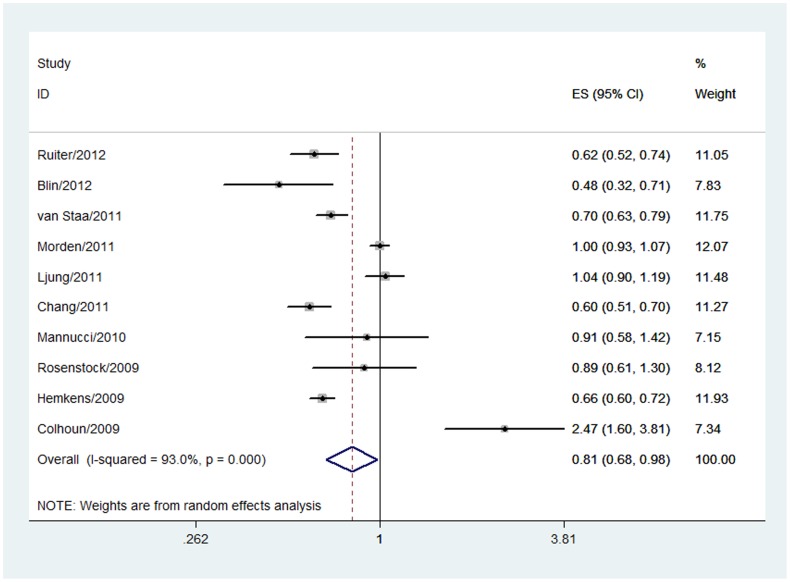
Forest plot evaluating the relationship between insulin glargine and overall cancer incidence.

Ten studies [7], [8], [23]–[25], [27]–[31] [6], [7], [14]–[16], [18]–[22] reported relative risk of insulin glargine and overall cancer. A pooled estimate of the 10 studies indicated that insulin glargine users had a significantly lower rate of overall cancer in comparison with non-glargine insulin users **(OR 0.81, 95% CI 0.68 to 0.98, P = 0.03**, Figure 2). In absolute terms, approximately 44 of every 1000 patients would fall cancer for non-glargine users and the use of insulin glargine can reduce this by 1 to 14 per 1000 patients. There was statistically significant heterogeneity **(P = 0.000, I^2^ = 93.0%).** The overall grade for the quality of evidence was very low (**Table 2**).

Then we performed a predefined subgroup analysis by comparators (including human insulin users, other insulin analogues users and insulin isophane users). Compared with other insulin analogues, insulin glargine use was associated with a lower **odds** of overall cancer in a random-effects model **(OR 0.76, 95% CI 0.62 to 0.93, P = 0.008), with significant heterogeneity (P = 0.003, I^2^ = 79.0%). The similar result was observed for insulin glargine users versus human insulin users (OR 0.64 95% CI 0.60 to 0.68, P = 0.000; p for heterogeneity = 0.410, I^2^ = 0%).** No significant difference was found in overall cancer for insulin glargine users versus insulin isophane users **(OR 0.67, 95% CI 0.43 to 1.07, P = 0.091)** in a random-effects model, with significant heterogeneity **(P = 0.02, I^2^ = 81%)**. To confirm the stability of the association of insulin glargine and overall cancer incidence, sensitivity analyses were conducted. When we limited to observational studies, the overall OR was **0.81 (95% CI 0.66–0.98, P = 0.03), with significant heterogeneity (P = 0.000, I^2^ = 94%). When we limited to cohort studies, the overall OR was 0.80 (95% CI 0.65–0.98, P = 0.03), with significant heterogeneity (P = 0.000, I^2^ = 95%). Exclusion of two studies by Morden et al.** [**27**] **and Colhoun et** ***al.*** **** [**7**] **in which not all patients were free of a history of cancer before cohort entry did not change the pooled estimate (OR 0.71, 95% CI 0.61 to 0.83, P<0.0001), with significant heterogeneity (P = 0.000, I^2^ = 84%). Exclusion of one study** [**30**] **that used self-reported data on insulin glargine therapy did not change the pooled estimate (OR 0.81, 95% CI 0.66 to 0.98, P = 0.03; p for heterogeneity = 0.000, I^2^ = 94%)**.

#### Insulin glargine and site-specific cancers incidence

**Figure 3:**
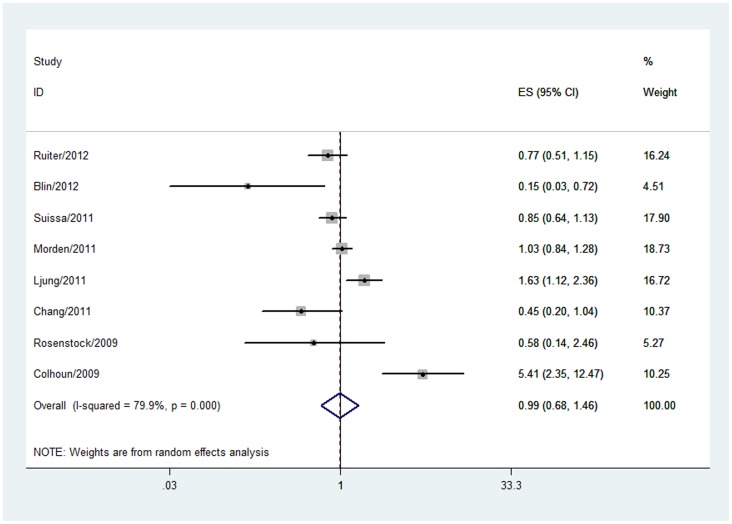
Forest plot evaluating the relationship between insulin glargine and breast cancer incidence.

Eight studies [7], [23], [24], [26]–[29], [31] including 284, 402 study subjects and 1, 364 breast cancer patients reported the of breast cancer in insulin glargine users. The overall OR for the eight studies was **0.99 (95% CI 0.68 to 1.46, P = 0.966**; very low-quality evidence) in a random-effects model for insulin glargine versus non-glargine insulin. A significant heterogeneity was detected **(P = 0.000, I^2^ = 79.9%,** **Table 2** **and** **Figure 3** **)**. In stratified analyses by study design [7], [23], [24], [26]–[29], the **odds** of breast cancer was not elevated with insulin glargine use compared to non-glargine insulin use in observational studies **(OR 1.02, 95% CI 0.68 to 1.53, P = 0.92; p for heterogeneity = 0.000, I^2^ = 83%). After removing two studies by Morden** ***et al.*** **** [**27**] **, and Colhoun** ***et al.*** **** [**7**] **in which not all patients were free of a history of any cancer before cohort entry, the overall outcome remained the same (OR 0.77, 95% CI 0.49 to 1.21, P = 0.26), with significant heterogeneity (P = 0.002, I^2^ = 73%).**

**In analysis of studies that reported the risk of gastrointestinal cancer, colorectal cancer, hepatobiliary cancer and bladder cancer in insulin glargine users, the overall ORs and corresponding 95% CIs were 0.70 (95% 0.51 to 0.95, P = 0.023), 0.69 (95% 0.56 to 0.85, P = 0.001), 0.51 (95% 0.37 to 0.70, P = 0.000) and 0.60 (95% 0.37 to 0.99, P = 0.046) in a random-effects model, respectively. When we conducted meta-analyses on the association between insulin glargine and other site-specific cancers, no evidence was found in an association of insulin glargine and prostate cancer (OR 0.94, 95% CI 0.63 to 1.42, P = 0.774), pancreatic cancer (OR 1.08, 95% CI 0.80 to 1.44, P = 0.627), and respiratory tract cancer (OR 0.91, 95% CI 0.59 to 1.41, P = 0.686). (** **Table 2** **)**

## Discussion

Findings of the meta-analysis indicated that compared with non-glargine insulin use, insulin glargine use was associated with **a 19% reduced odds** of overall cancer in patients with diabetes. Results were consistent in subgroup analysis and sensitivity analysis.

Similar results were found in a combined analysis [32] of 31 randomized trials, notwithstanding the summary analysis of data was limited by its sample size and most studies included in the combined analysis were of 6 months' duration. Recently, a randomized controlled trial comparing insulin glargine use with standard care was published in New England. In this trial, a total of 12,537 people with cardiovasclar risk factors plus impaired fasting glucose, impaired glucose tolerance, or patients with type 2 diabetes were randomly assigned to receive insulin glargine or standard care and to receive n–3 fatty acids or placebo. The trial lasted for 6.2 years and their data did not support the relationship between insulin glargine and the risk of incident cancers [33]. In 2002, an animal study [34] that lasted for 2 years demonstrated that insulin glargine did not have a systemic cancerigenic potential in rats and mice. A recent study [35] by the same investigators reevaluating the carcinogenicity potential of insulin glargine indicated that cancer risk was found to be no greater for animals treated with insulin glargine than for the control-treated animals. They considered that insulin glargine was not likely to pose a cancer risk in humans and the findings needed to be confirmed by ongoing clinical studies. Their conclusion is consistent with our meta-analysis. In a study [36] using human follicular thyroid cancer cell line FTC-133, insulin glargine displayed similar mitogenic potency in comparison with human insulin. In several studies [13]–[15] using non-malignant cells, insulin glargine showed a similar mitogenic potency compared to human insulin. These studies may be as an evidence to support our results. Our results indicated that compared with non-galrgin insulin, insulin glargin did not increase the overall cancer incidence, but decreased the **odds** of overall cancer.

Compared with non-glargine insulin, Insulin glargine use was associated with lower **odds** of **gastrointestinal cancer, colorectal cancer, hepatobiliary cancer and bladder cancer**. No significant association was found between insulin glargine and **other site-specific cancer.**

The association of insulin glargine and breast cancer was wildly inconsistent in different studies. Three of the included studies [7], [23], [28] reported an increased risk of breast cancer in insulin glargine users, three studies [26], [27], [29], [31] showed that glargine was not associated with significantly increased risk of breast cancer measure and one study [24] reported a lower risk of breast cancer. Suissa *et al.* [26] found that the insulin glargine use was not associated with an increased risk of breast cancer during the first 5 years, but longer-term use may increase the risk. Suissa *et al.* considered two non-mutually exclusive mechanisms of insulin [26]. One mechanism involves a stimulatory effect of insulin on the growth rate of the breast cancer that are present not yet of a size that can be diagnosed. This mechanism generates a relatively short-term effect (evident in under 2 years), similar to the effects of postmenopausal hormone replacement therapy on breast cancer risk. The other mechanism involves an effect on the process of gradual carcinogenesis (accumulation of genetic damage resulting in transformation) where the related receptors are on mammary epithelial cell. Insulin is hypothesized to promote stepwise carcinogenesis due to long-term exposure. Several experimental studies [11], [12], [37] showed that insulin glargine promoted the proliferation of breast adenocarcinoma cell in vitro. These data were considered to be a plausible explanation for the increased breast cancer. However, Staiger *et al.* found that there was no evidence that insulin glargine and regular insulin differ in their mitogenic potency in nomal and transformed breast epithelial cell [38]. Similar outcome was reported in breast cancer cell line MCF-7 cells that had the highest expression of IGT-I receptor [39]. Moreover, many researchers thought the hypothesis that insulin glargine is more mitogenic than non-glargine in vivo remained unproven. So we couldn't excluded the possibility that glargine was associated with an increased risk. An experimental study [40] showed that there was no significant difference between glargine and regular human insulin concerning regulation of proliferation and apoptosis of human pancreatic cancer cells. In an animal study [41], investigators found that insulin glargine did not increase cell proliferation compared with insulin isophane in healthy colonic mucosa of diabetic rats. Though their data cannot be directly extrapolated to humans, yet they supported our results as evidence.

**Similar to our result, Boyle et al.** [**42**] **and Du et al.** [**43**] **reported that the use of insulin glargine did not increase the incidence of cancer. There are some differences between the present meta-analysis and the previous ones. First, the study by Boyle et al that only included 8 studies is a conference paper and the full text hasn't been published up to now. The study did not observe the association between insulin glargine and site-specific cancer incidence. Second, the study by Du et al only included 7 studies. Of these included studies, two studies included the same population (the study by Ljung et al and the study by Jonasson et al) and one study** [**44**] **is a meta-analysis, whereas its inclusion criteria were original studies in cohort studies design. Finally, though both the present analysis and the previous ones found that the use of insulin glargine did not increase the incidence of cancer, yet the present study reported a decreased incidence of overall cancer and some site-specific cancer (gastrointestinal cancer, colorectal cancer, hepatobiliary cancer and bladder cancer).**

The strengths of our review include the comprehensive meta-analysis with a comprehensive search strategy, rigid inclusion criteria, methodological quality assessments using the GRADE system and detailed assessment of the factors that influence the confidence in the results across questions and studies. In addition, by integrating the actual evidence, our meta-analysis allowed a more objective appraisal of the literature by resolving uncertainty when the original study data did not agree.

Several potential limitations should be considered. First, **there existed significant heterogeneity in terms of population demographics, follow-up time, study design, and insulin dose. We are not able to account for these differences, despite the fact that proper meta-analytic methodology with random-effects models was used and that different sensitivity analyses were carried out.** Second, some of the included studies **did not** distinguish between type 1 and type 2 diabetes which may influence any true relation. **Third, metformin was considered as a protective reagent against the development of some cancers** [**45**] **, but few studies included in the present meta-analysis controlled for the effect of metformin which may influence the results. Fourth, the follow-up period of most of included studies is very short which may influence true results. Fifth, more and more studies showed that diabetic individuals have an increased risk of cancer compared to non-diabetic individuals. But only one study included in the present meta-analysis adjusted for diabetes, so we are not able to further perform a meta-analysis to observe whether diabetes itself influence the effect.** Finally, the incidence of breast cancer differs for premenopausal and postmenopausal women. However, most of studies did not distinguish between premenopausal and postmenopausal women.

In conclusion, the meta-analysis provides no evidence that insulin glargine use is positively associated with overall cancers and site-specific cancers compared with non-glargine. It seems that these findings reassure most glargine users. However, the association between insulin glargine and breast cancer requires further investigations.

## Supporting Information

Data S1 **Literature search strategy.**
(DOC)
