# Supplementary material for: Insulin Glargine and Cancer Risk in Patients with Diabetes: A Meta-Analysis
Source: PLoS One. 2012 Dec 19;7(12):e51814. doi: 10.1371/journal.pone.0051814 (PMC3526637; doi:10.1371/journal.pone.0051814)
Supplement: Data S1 — Literature search strategy. (DOC) [file pone.0051814.s001.doc]

**Literature Search Strategy with relevant keywords**

glargine (1), insulin glargine (2), Lantus(3), Tumors (4), Tumor (5), Cancer (6), Cancers (7) Neoplasms (8), Neoplasm (9), malignancy (10), maligancies (11)

Combination search strategy: [TERMS 1 OR 2 OR 3] and [TERMS 4 OR 5 OR 6 OR 7 OR 8 OR 9 OR 10 OR 11].
